# Predicting Soluble Nickel in Soils Using Soil Properties and Total Nickel

**DOI:** 10.1371/journal.pone.0133920

**Published:** 2015-07-28

**Authors:** Xiaoqing Zhang, Jumei Li, Dongpu Wei, Bo Li, Yibing Ma

**Affiliations:** 1 School of Forestry and Horticulture, Hubei University for Nationalities, 39 Xueyuan Road, Enshi, Hubei Province, 445000, China; 2 Institute of Agricultural Resources and Regional Planning, Chinese Academy of Agricultural Sciences, 12 Southern Street of Zhongguancun, Beijing, 100081, China; 3 School of Resources and Environment, University of Jinan, Jinan, 250022, Shandong Province, China; Old Dominion University, UNITED STATES

## Abstract

Soil soluble nickel (Ni) concentration is very important for determining soil Ni toxicity. In the present study, the relationships between soil properties, total and soluble Ni concentrations in soils were developed in a wide range of soils with different properties and climate characteristics. The multiple regressions showed that soil pH and total soil Ni concentrations were the most significant parameters in predicting soluble Ni concentrations with the adjusted determination coefficients (R_adj_^2^) values of 0.75 and 0.68 for soils spiked with soluble Ni salt and the spiked soils leached with artificial rainwater to mimic field conditions, respectively. However, when the soils were divided into three categories (pH < 7, 7–8 and > 8), they obtained better predictions with R_adj_^2^ values of 0.78–0.90 and 0.79–0.94 for leached and unleached soils, respectively. Meanwhile, the other soil properties, such as amorphous Fe and Al oxides and clay, were also found to be important for determining soluble Ni concentrations, indicating that they were also presented as active adsorbent surfaces. Additionally, the whole soil speciation including bulk soil properties and total soils Ni concentrations were analyzed by mechanistic speciation models WHAM VI and Visual MINTEQ3.0. It was found that WHAM VI provided the best predictions for the soils with pH < 7, was relatively reasonable for pH 7 to 8, and gave an overestimation for pH > 8. The Visual MINTEQ3.0 could provide better estimation for pH < 8 and meanwhile quite reasonable results for pH > 8. These results indicated the possibility and applicability of these models to predict soil soluble Ni concentration by soil properties.

## Introduction

Soil soluble metals are considered to be most active fractions for bioavailability/toxicity to soil organisms, and are governed by soil chemistry and the solid/solution distribution of total metals. Dominant soil chemical/physical properties known to affect the bioavailability of contaminants include: soil pH, soil organic matter (SOM), clay, reactive iron, aluminum, and manganese oxides [1]. Research has shown that empirical and mechanistic models can provide accurate estimates of soluble metal concentrations. Most empirical estimate values are derived from multiple regression analyses of soil physicochemical properties and total soil metal concentrations [2–4]. However, integration of a semi-mechanistic model (based on the competitive adsorption of metal) into the multiple regression analyses has been proven to be more successful at predicting soluble metal concentrations [3, 5–6]. In this study, various sorption surfaces were taken into account, not only the soil organic matter, but also clay silicates and metal oxides.

As for mechanistic models, WHAM VI has been widely used for metal speciation in soil systems [2, 7–10]. At present, few studies have used the whole soil speciation approach to predict soluble metal concentration [2, 11]. Thakali et al. [11] used bulk properties and total Ni and SOM content as inputs in WHAM VI to predict dissolved Ni^2+^ concentrations with a root mean square error (RMSE) of 0.39 for soils with a pH < 7 and a CaCO_3_ content of nearly 0. However, the ability of WHAM VI to conduct whole soil speciation in calcareous soils characterized by high pH (pH > 7) and significant CaCO_3_ content, representative of certain types and properties of Chinese soils, appears not to have been attempted. In the present study, we attempted to use the same whole soil approach to compute Ni speciation in laboratory-spiked soils.

Visual MINTEQ 3.0 is another well-known mechanistic model, and has been increasingly employed for calculating soluble metal speciation [12–14]. Most of these attempts were performed on soluble phases (natural water, solutions, and pore water), whereas use tests of whole soil approaches are rare. The Stockholm Humic Model (SHM) in Visual MINTEQ3 was specified to calculate cation binding to humics, a reaction that provides more realistic assessment of metal–humic complexation, and also can be used to calculate both dissolved and solid humic substance bindings [15]. According to previous studies, SHM has been proven to better describe proton binding and dissolved metal concentrations [13, 16]. However, the application of this model for speciation of soil soluble Ni still requires further investigation.

The objectives of the present study were to: (i) investigate the influence of major soil properties on Ni solid–solution distributions; (ii) develop competitive adsorption models that use multiple regressions to predict soil soluble Ni concentrations in 17 typical Chinese soils with a wide range of soil properties and total metal concentrations; and (iii) attempt to apply the speciation models (WHAM VI and Visual MINTEQ3) for whole soil speciation and to determine whether they can provide reasonable predictions.

## Materials and Methods

### 2.1. Soil Sampling and Treatment

The present study was carried out on 17 soil samples, which covered the main areas of China (Table 1) and represented the major types of Chinese agricultural soils. These soils were described in full in a separate paper [17]. The ranges of soil properties were as follows: pH 4.93–8.90; organic carbon content (OC) 0.60%–4.28%; and clay content 10%–66%.

**Table 1:** The locations and main properties of soils used in the present study.

No	Location	Soil type	pH	EC(uS/cm)	eCEC(cmol/kg)	TC(%)	OC(%)	Clay(%)	Silt(%)	Sand(%)	Al_ox_^1^(mg/kg)	Fe_ox_(mg/kg)	Mn_ox_(mg/kg)
**1**	Lingshan, Beijing (39°55'N116°8'E)	Brown earth	7.48	92.5	22.6	4.8	4.3	20	21	59	1304	1697	267
**2**	Beipei, Chongqing (30°26'N106°26'E)	Purplish soil	7.12	71	22.3	1.0	1.0	27	25	48	603	989	283
**3**	Zhangye, Gansu (38°56'N100°27'E)	Irrigated desert soil	8.86	151.8	8.08	1.9	1.0	20	24	56	674	1980	233
**4**	Guangzhou, Guangdong (23°10'N113°18'E)	Paddy soil	7.27	136.7	8.30	1.5	1.5	25	12	62	532	1811	33
**5**	Hailun, Helongjiang (47°28'N126°57'E)	Black soil	6.56	153	33.6	3.0	3.0	40	27	33	1954	3298	451
**6**	Haikou, Hainan (19°55'N111°29'E)	Latersol	4.93	110.8	8.75	1.5	1.5	66	18	16	1736	1337	200
**7**	Hangzhou, Zhejiang (30°26'N120°25'E)	Paddy soil	6.80	203.3	12.83	2.5	2.5	39	36	25	1003	4980	135
**8**	Qiyang, Hunan (26°45'N111°52'E)	Red earth	5.31	74.1	7.47	0.9	0.9	46	35	19	1326	1146	294
**9**	Jiaxing,Zhejiang (30°77'N120°76'E)	Paddy soil	6.70	158.8	19.3	1.4	1.4	41	42	17	1106	6212	261
**10**	Gongzhuling, Jilin (42°40'N124°88'E)	Black soil	7.82	146.9	28.7	2.2	2.2	45	26	29	1786	1447	387
**11**	Langfang, Hebei (39°31'N116°44'E)	Fluvo-aquic soil	8.84	5.70	6.36	0.9	0.6	10	4	86	291	537	74
**12**	Hulunber, Neimeng (46°03'N22°03'E)	Chernozem	7.66	888	22.7	2.7	2.7	37	16	47	1441	2477	307
**13**	Dezhou, Shandong (37°20'N116°29'E)	Fluvo-aquic soil	8.90	111.8	8.33	1.4	0.7	18	18	64	497	644	145
**14**	Yanglin, Shanxi (34°19'N108°0'E)	Loessial soil	8.83	83.2	8.46	1.7	0.6	27	41	31	863	707	288
**15**	Shijiazhuang, Hebei (38°03'N114°26'E)	Cinnamon soil	8.19	302	11.7	1.5	1.0	21	22	57	734	826	222
**16**	Urumchi, Xinjiang (43°95'N87°46'E)	Gray desert soil	8.72	226.5	10.3	1.5	0.9	25	23	52	551	600	251
**17**	Zhengzhou, Henan (34°47'N112°40'E)	Fluvo-aquic soil	8.86	108.7	8.50	1.6	1.6	16	13	70	482	581	121

EC: electric conductivity; eCEC: effective cation exchange capacity; TC: total carbon; OC: organic carbon; ^1^ox: oxalate extractable metal.

Soil samples were spiked with NiCl_2_ solution with a range of eight concentrations from 37.5 to 2400 mg Ni/kg for soils with pH > 7; 25 to 1600 mg Ni/kg for soils with pH 5 to 7; and 12.5 to 800 mg Ni/kg for soils with pH < 5, respectively. The eight different amended soil samples for each site were leached with artificial rainwater to overcome potential salinity effects and to reduce the difference in Ni speciation between laboratory-treated and field-aged soils, and for comparison of the Ni partition between unleached soils [17–18]. The spiked soil samples were stored for at least two months before measurement of soil and pore water properties. None of the soil samples before amendment with Ni were contaminated by other heavy metals (Cu, Zn, Pb, As, Cd and Cr), the exception being the total Ni concentration in the Haikou, Hainan soil sample (No. 6 in Table 1), which was as high as 124 mg/kg.

### 2.2. Analyses of Soil and Soil Solutions

Soil pH and electrical conductivity (EC) were determined in a water suspension of soil using a 1:5 (soil:solution) ratio. The main soil properties were determined, including: texture, effective cation exchange capacity, OC, total carbon content, and total Ni content, according to Li et al. [17]. The concentrations of amorphous iron, manganese, and aluminum in the soils were extracted by ammonium oxalate extraction and analyzed using an inductively coupled plasma-optical emission spectrometer (ICP-OES) (SpectroFlame Modula, Spectro, Boschstr, Kleve, Germany).

Soil solutions were extracted by centrifuge for 45 minutes at a speed of 3500 r/min and then another 45 minutes at a higher speed of 15000 r/min after incubation overnight at 50 cm water tension and 20°C. The extracted soil pore water was passed through 0.45μm filters. The dissolved organic carbon (DOC), pH, EC, Na^+^, K^+^, Ca^2+^, Mg^2+^ and Ni^2+^ in the soil pore water samples were measured immediately. DOC was analyzed using the Formacs SERIES TOC/TN Analyzer (Skalar Ltd., Breda, the Netherlands). The major ions (K^+^, Ca^2+^, Na^+^, Mg^2+^, Ni^2+^) were measured either by ICP-OES or inductively coupled plasma-mass spectrometry (SpectroflameModula, Spectro, Boschstr, Kleve, Germany), depending on their concentrations [19]. Pore water properties for 136 soil samples (17 site samples with eight Ni concentration levels) were determined for both unleached and leached treatments. Some soluble Ni concentrations were less than the detection limits, only 102 and 97 data sets were adopted for further analyses and modeling for unleached and leached soil samples, respectively.

### 2.3. Competition and the Adsorption Model

The partitioning of Ni between the solid and the solution phase was determined by soil properties, such as pH, clay, soil organic matter, metal oxides/hydroxides (Al, Fe and Mn). Eq 1, which was derived from the empirical Freundlich model [3, 6], was employed to describe how Ni competes with cations for binding and exchange sites. The soluble Ni concentration in soil pore water (Ni_dis_), total Ni in soil (Ni_tot_), and the important soil properties were logarithms of values.

$$\begin{array}{l} N{\text{i}}_{dis}=\mathrm{lg}Ni_{tot}+pH+\mathrm{lg}OC+\text{lg}\;Clay+\mathrm{lg}Fe_{ox}+\ldots \end{array}$$ (1)

### 2.4. Soil Speciation Models

Two chemical mechanism calculation models (WHAM/Model VI and Visual MINTEQ version 3.0) were employed to estimate the soluble Ni concentration in the soil solution. These two models were used with the default parameters. The required bulk soil properties used as input data were: total concentration of Ni; SOM content; DOC concentrations; cations (K^+^, Na^+^, Mg^2+^, Ca^2+^); estimated solution concentration of Cl^-^ and SO_4_^2-^; soil solution pH; and activity concentration of Fe^3+^ and Al^3+^. The solubility of Al (OH)_3_, Fe (OH)_3,_ and the active fraction of DOC were included to optimize the prediction of soluble Ni concentrations. For WHAM VI, humic substances were presumed to be the only ion-binding components of the soil–solid phase. Other studies have shown that other soil phases, such as clays and metal oxides, may also contribute to metal sorption [2]. Therefore, clay and metal oxide were also included to improve the fit.

In MINTEQ, the SHM model was similar to WHAM VI in many aspects and thus the input data was consistent with those used in WHAM VI. In some soil samples, the soil solution may become oversaturated when the pH is more than 7 and the CaCO_3_ content above 0. Some potential solid phases (NiCO_3_ (lgK_s_ = –11.2)) were specified, and if these solids did not physically exist they did not affect the equilibrium. The details of the speciation procedures of WHAM VI have been described in detail by Tipping et al. [2], Thakali et al. [11], and Sjöstedt et al. [13].

## Results, Discussion, and Conclusions

### 3.1. Multiple Regression Models

**Fig 1:**
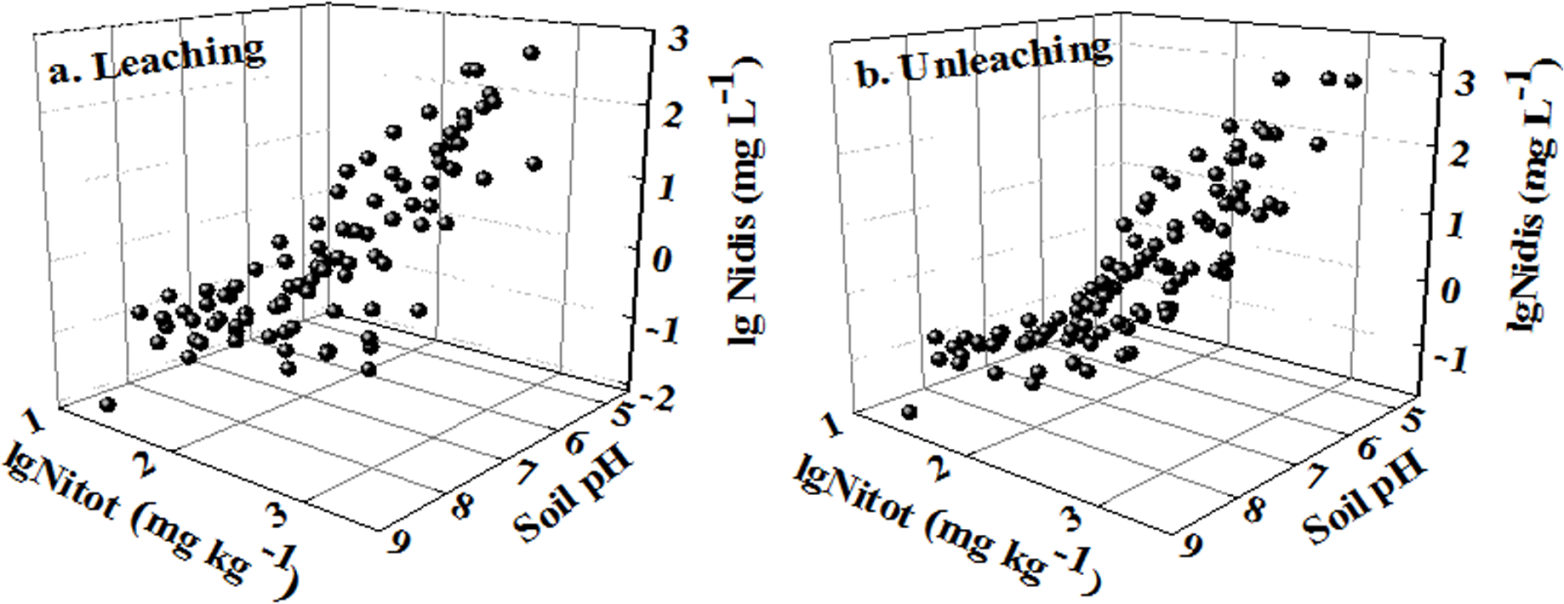
Soluble Ni concentrations as a function of total Ni concentrations and soil pH (Ni_tot_ and Ni_dis_ represented total Ni concentration in soil and the soluble Ni concentration in soil pore water, respectively).

Multiple regression analysis was performed to derive the relationships between the soluble Ni concentrations in pore water, total soil Ni concentrations, and soil properties (lg transformed) (Table 2). These selected soil properties were significantly correlated with soluble Ni concentrations, which were evaluated through Pearson correlation analysis (S1 and S2 Tables in Supporting Information). The regression results showed that the total concentration of Ni and pH were the most important factors controlling the soluble Ni concentration in pore water for the whole pH range, with the R_adj_^2^ of 0.75 for soils spiked with soluble Ni salt, and 0.68 for the spiked soils leached with artificial rainwater to mimic field conditions (Table 2). Soluble Ni concentrations were strongly controlled by soil Ni concentrations and soil pH, meaning that Ni_dis_ increased when Ni_tot_ increased and pH decreased (Fig 1), indicating competitive adsorption between H^+^ and Ni for soil binding sites. Previous studies also showed that soil pH was the most important factor influencing Ni partitioning in soils [3, 6]. Compared with pH, other soil factors exhibited relatively weak effects on soluble Ni concentration. Incorporation of amorphous Al_ox_ slightly improved the prediction in leached soils, and was identical with clay for unleached soils (equations 3 and 17 in S3 Table).

**Table 2:** Multiple regressions between log Ni_dis_ (soluble Ni concentration in soil pore water) and log Ni_tot_ (total Ni in soil) together with soil properties.

pH	No.	Regression equations	n	R^2^	R_adj_^2^	P
**Leaching**						
**all**	1	lgNi_dis_ = 0.76+1.24 lgNi_tot_-0.46 pH	97	0.69	0.68	***
**<7**	2	lgNi_dis_ = 20.33+1.85 lgNi_tot_-4.53lgAl_ox_-2.82lgFe_ox_	30	0.90	0.88	***
**7~8**	3	lgNi_dis_ = -1.53+1.84lgNi_tot_-0.87lgAl_ox_	26	0.91	0.90	**
**>8**	4	lgNi_dis_ = 5.66+0.79lgNi_tot_-0.53pH-3.22lgAl_ox_+1.94lgFe_ox_	41	0.80	0.78	*
**Unleaching**						
**all**	5	lgNi_dis_ = -0.24+1.51lgNi_tot_-0.39pH	102	0.75	0.75	***
**<7**	6	lgNi_dis_ = 37.83+2.0lgNi_tot_-4.25pH+5.01lgFe_ox_-20.03lgClay	32	0.89	0.88	***
**7~8**	7	lgNi_dis_ = 5.70+2.10lgNi_tot_-1.39pH	28	0.95	0.94	***
**>8**	8	lgNi_dis_ = -3.45+1.11lgNi_tot_+0.85lgFe_ox_-1.33lgClay	42	0.80	0.79	**

Ni_dis_: soluble Ni concentration in soil pore water; Ni_tot_: total Ni concentration in soil; R^2^: coefficient of determination; R_adj_^2^: adjusted coefficient of determination; p: significant level of factors in regression equations; *: 5% significant level

**: 1% significant level

***: 1‰ significant level.

In order to better examine the influence of soil properties on soluble Ni concentration, the selected soil samples were segregated into three categories: acid (pH < 7), weak alkaline (7 < pH < 8) and strong alkaline (pH > 8), in accordance with the soluble Ni speciation distribution pattern [20]. Regression models based on these three pH ranges provided significantly better fits: the R_adj_^2^ increased to more than 0.79, and was especially higher in the acid and weak alkaline soils, and the predictions were within a half order of magnitude of the measured ones (S1 and S2 Figs). For soil pH between 7 and 8, Al_ox_ was significantly related with Ni_dis_ in leached soils, whereas a significant effect of pH was observed in unleached soils (equations 3 and 7 in Table 2). For the rest of the soils, amorphous Al, Fe oxide, and clay also demonstrated significant affinity with soluble Ni, indicating that Ni sorption onto the soils was influenced by their presence. However, the significant factors observed in leached soils did not completely match those in unleached soils. On one hand, leaching treatments partially changed soil salinity and pH and consequently influenced the soluble Ni concentration. On the other hand, the soil properties themselves were intercorrelated. For example, Al_ox_ was closely connected with pH and clay content (S1 and S2 Tables). Thus, the influence of Al_ox_ on soluble Ni content in leached soil may indirectly reflect the effect of pH and soil clay content, which could explain the differences between equations 2 and 4 and between 6 and 8 (Table 2). Meanwhile, the predictions were slightly improved when all of the factors were included in the multiple regressions (S3 Table).

For leached soils, a negative relationship was observed between Al_ox_ and soluble Ni concentration. A number of studies have demonstrated that the high affinity of Al_ox_ for metal is due to large surface areas, microporous structures, and an abundance of binding sites [21]. In unleached soils, amorphous Fe_ox_ was positively correlated with soluble Ni concentrations, which was not consistent with previous studies [6]. The solid and dissolved amorphous Fe_ox_ in the soil was mainly in the forms of oxides and hydroxides, respectively. Dissolved colloidal Fe hydroxides could form complexes with Ni and DOC complexes [22], which influenced soluble Ni speciation and potentially increased Ni mobility. Additionally, adsorption of humic matter altered the surface chemistry and colloidal stability of iron oxides. In the case of DOC, they would compete with Ni for the iron oxide surface sites, and consequently, DOC occupied a portion of all surface sites. As for Ni, the soil absorption capacity decreased relatively and more Ni partitioned to the soluble phase. Therefore, the soluble Ni concentration depended on the partition of Fe_ox_ in solid and solution phases.

### 3.2. WHAM Speciation for Soluble Ni Concentration

The precision in the soluble Ni speciation calculation depended on the input data of total concentrations of soil parameters. According to previous models [2, 11], several inputs were considered to optimize the prediction, including: (1) the adsorptive surfaces of clay and amorphous Al, Fe, and Mn oxides; (2) the solubility products of Fe^3+^ and Al^3+^; (3) the fraction of active SOM and DOC; and (4) the dissolved cations (K^+^, Na^+^, Mg^2+^ and Ca^2+^). The inputs list and modeled optimized results are presented in Table 3.

**Table 3:** Effects of the input variables on the RMSE between the predicted soluble Ni concentrations by WHAM and measured values. (The temperature is set at 293K and the partial pressure of CO_2_ (pCO_2_) is set at 10^−3.5^ atm).

No.	WHAM VI Inputs	RMSE (Leaching)				RMSE (Unleaching)			
		all	<7	7–8	>8	all	<7	7–8	>8
**1**	Ni_tot_+SOM+DOC(65%AFA)+pH+Cation (K^+^, Na^+^, Ca^2+^, Mg^2+^)+anion (Cl^-^, SO_4_^2^)^-^	1.2	0.41	0.74	1.80	1.3	0.37	0.6	1.83
**2**	Ni_tot_+SOM+DOC(65%AFA)+pH+Cation (K^+^, Na^+^, Ca^2+^, Mg^2+^)+anion (Cl^-^, SO_4_^2^)^-^+Fe_ox_	1.1	0.35	0.68	1.74	1.1	0.33	0.55	1.77
**3**	Ni_tot_+SOM+DOC(65%AFA)+pH+Cation (K^+^, Na^+^, Ca^2+^, Mg^2+^)+anion (Cl^-^, SO_4_^2^)^-^+Fe_ox_+Al_ox_	1.1	0.35	0.67	1.72	1.1	0.34	0.51	1.75
**4**	Ni_tot_+SOM+DOC(65%AFA)+pH+Cation (K^+^, Na^+^, Ca^2+^, Mg^2+^)+anion (Cl^-^, SO_4_^2^)^-^+Fe_ox_+Al_ox_+Mn_ox_	1.1	0.34	0.66	1.71	1.1	0.33	0.50	1.74
**5**	Ni_tot_+SOM+DOC(65%AFA)+pH+Cation (K^+^, Na^+^, Ca^2+^, Mg^2+^)+anion (Cl^-^, SO_4_^2^)^-^+Fe^3+^ (${p\;K}_{{Fe(OH)}_{3}}$ = 3.0)	1.2	0.45	0.76	1.82	1.2	0.41	0.64	1.84
**6**	Ni_tot_+SOM+DOC(65%AFA)+pH+Cation (K^+^, Na^+^, Ca^2+^, Mg^2+^)+anion (Cl^-^, SO_4_^2^)^-^+Fe^3+^ (${p\;K}_{{Fe(OH)}_{3}}$ = 3.0) +Al^3+^ (${p\;K}_{{A\text{l}(OH)}_{3}}$ = 6.0)	1.2	0.46	0.77	1.83	1.2	0.44	0.66	1.85
**7**	Ni_tot_+SOM+DOC(65%AFA)+pH+Cation (K^+^, Na^+^, Ca^2+^, Mg^2+^)+anion (Cl^-^, SO_4_^2^)^-^+Fe_ox_+Clay	1.1	0.35	0.66	1.69	1.1	0.31	0.53	1.74
**8**	Ni_tot_+SOM+DOC(30%AFA)+pH+Cation (K^+^, Na^+^, Ca^2+^, Mg^2+^)+anion (Cl^-^, SO_4_^2^)^-^+Fe_ox_+Clay	1.1	0.29	0.66	1.67	1.1	0.30	0.51	1.73
**9**	Ni_tot_+SOM+pH+Cation (K^+^, Na^+^, Ca^2+^, Mg^2+^)+anion (Cl^-^, SO_4_^2^)^-^+Fe_ox_ +Clay	1.1	0.29	0.66	1.66	1.1	0.31	0.50	1.72
**10**	Ni_tot_+SOM+pH+Cation (Na^+^, Ca^2+^, Mg^2+^)+anion (Cl^-^, SO_4_^2^)^-^+Fe_ox_+Clay	1.1	0.29	0.66	1.66	1.1	0.31	0.50	1.72
**11**	Ni_tot_+SOM+pH+Cation (Ca^2+^ and Mg^2+^)+anion (Cl^-^, SO_4_^2^)^-^+Fe_ox_ +Clay	1.1	0.29	0.66	1.66	1.1	0.31	0.49	1.72
**12**	Ni_tot_+SOM+pH+Cation (Ca^2+^)+anion (Cl^-^, SO_4_^2^)^-^+Fe_ox_+Clay	1.0	0.30	0.68	1.60	1.1	0.31	0.45	1.66

Ni_tot_: total Ni concentration in soil; SOM: soil organic matter; DOC: dissolved organic carbon; AFA: active fulvic acid; Al_ox_: amorphous Al oxide; Fe_ox_: amorphous Fe oxide; Mn_ox_: amorphous Mn oxide; RMSE: root mean square error.

The inclusion of Fe oxides improved the prediction with a small decrease in RMSE values (no. 1 to 2 in Table 3), indicating their contribution to Ni adsorption. Meanwhile, Al and Mn oxides showed insignificant effects on Ni sorption. For example, in unleached soils with pH < 7, the RMSE values were very similar (both at 0.33) when the inputs were Al and Mn oxides (no. 2 to 4 in Table 3). These results could be explained by the comparative strong fixation of Fe oxides on soil Ni [23–24]. With inclusion of clay content, a slight improvement in prediction was found in a comparison of no. 2 and 7 in Table 3. Similar observations were obtained by Shi et al. [25], where Ni binding to clay minerals was relatively small in comparison with SOM. The small contribution of clay to metal binding may be due to the relatively low metal loading [26].

The activities of Fe^3+^ and Al^3+^ were determined by their hydrolysis and interaction with Ni for binding sites. The optimized solubility constants were referenced from Thakali et al. [11] with ${\text{p K}}_{{\text{Fe}(\text{OH})}_{3}}$ = 3.0 and ${\text{p K}}_{{\text{Al}(\text{OH})}_{3}}$ = 6.0, respectively. However, the inclusion of Fe and Al activity did not improve the prediction and conversely aggravated the differences between the predicted and observed concentrations (no. 5 and 6 in Table 3).

SOM was assumed to be an important adsorbent due to its high affinity to Ni and the assessment of active fraction of SOM was essential in Ni speciation. According to previous research, SOM consisted of particulate FA and HA with a ratio of 84:16 [27]. Earlier publications defined 65% of DOC as colloidal FA [2, 11], and others were assumed inert. In the present study, results were over predicted when the active fraction of DOC was 65%. Adjusting the active fraction to 30%, the RMSE values were smaller than those with 65% (no. 7 and 8 in Table 3). Further decreasing the fraction to 0%, the predicted results were similar to those with a 30% active fraction of DOC, indicating that DOC may be neglected in predicted dissolved Ni concentrations.

When considering the dissolved cations, K^+^ and Na^+^ did not affect the precision of prediction (no. 9 to 11 in Table 3). The exclusion of dissolved Mg^2+^ resulted in a fluctuation of RMSE in different pH ranges with the RMSE nearly unchanged for soils with pH < 7 and decreased for the rest of the soils (no. 11 to 12 in Table 3). The effects of Mg could not be ignored in determining soluble Ni concentration for soils with pH > 7; nevertheless dissolved Ca^2+^ was deemed essential as the only dissolved cation that improved the prediction in some soils.

**Fig 2:**
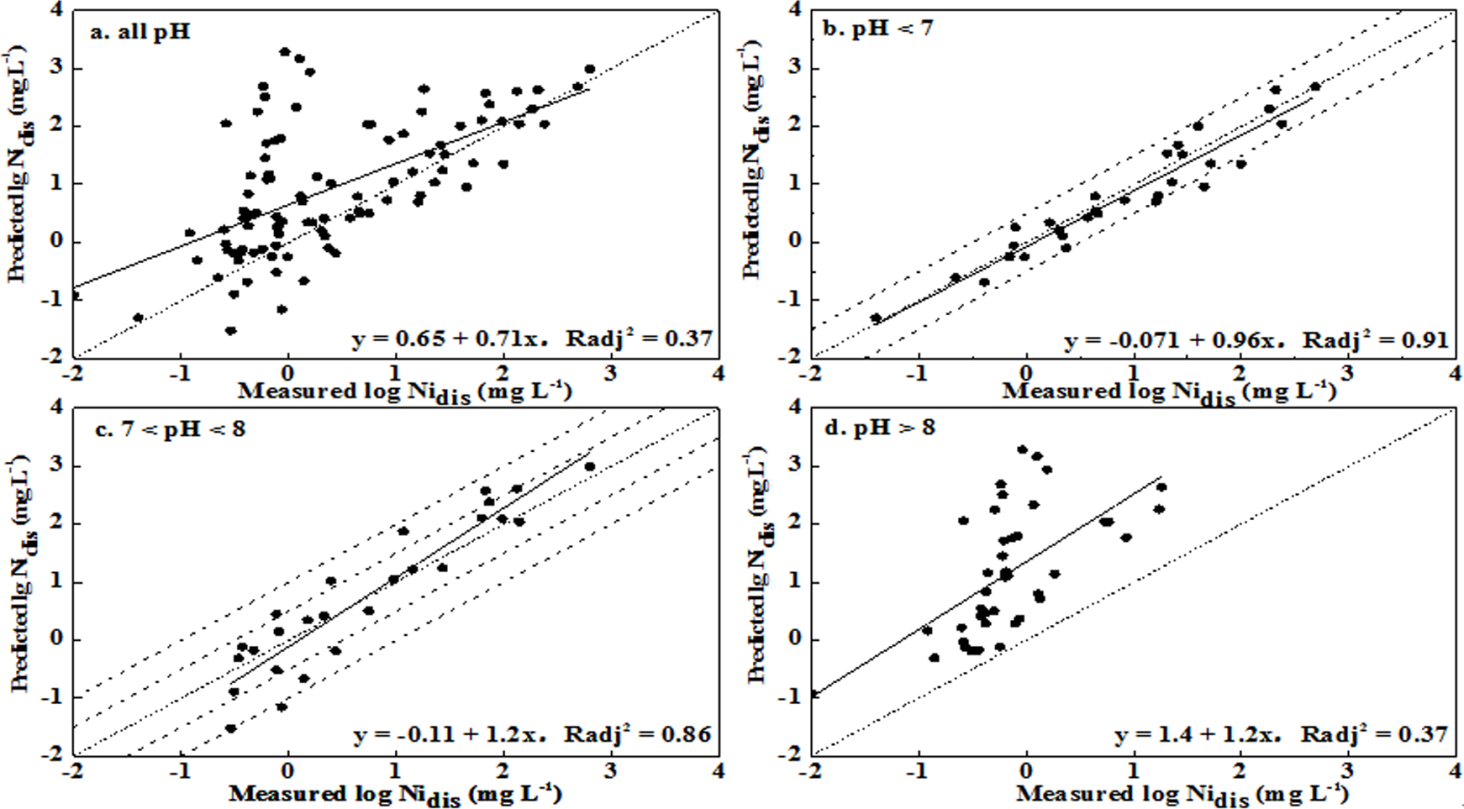
Measured soluble Ni concentrations versus predicted Ni concentrations using WHAM VI for leached soils (Ni_dis_ represented the soluble Ni concentration in soil pore water).

**Fig 3:**
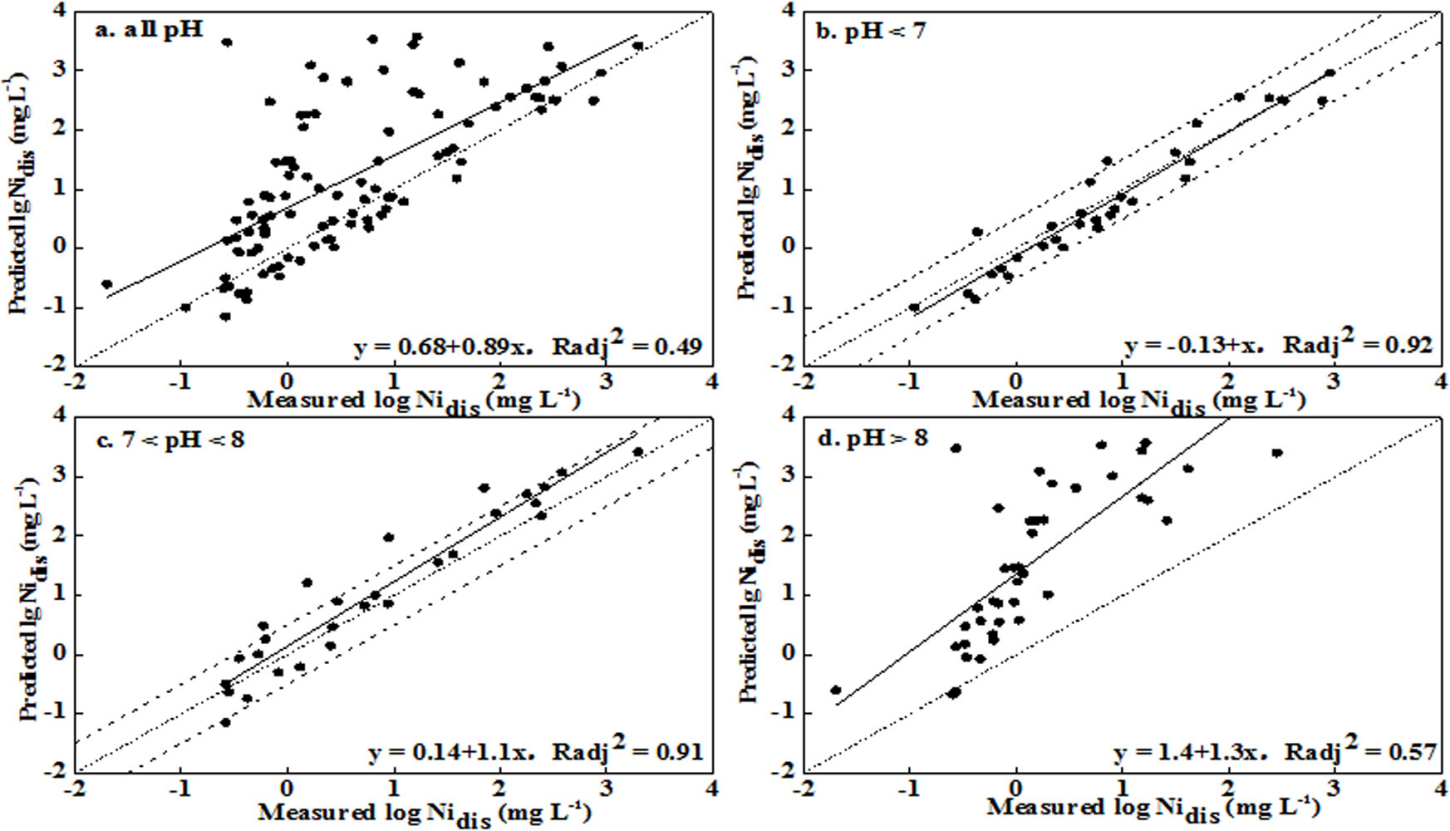
Measured soluble Ni concentration versus predicted Ni concentration using WHAM VI in unleached soils (Ni_dis_ represented the soluble Ni concentration in soil pore water).

The above analyses show that the optimized and simplified parameters of the inputs to WHAM VI included: total Ni, SOM, pH, Fe_ox_, Clay, dissolved Ca^2+^, Cl^-^, and SO_4_^2-^. For the unleached soils, WHAM VI could provide better predictions for soils with pH < 8 and weaker predictions for soils with pH > 8. The RMSE values were 0.31, 0.45, and 1.66 in the three pH ranges (< 7, 7–8, and > 8), corresponding to deviations of 2.0-, 2.8-, and 45.8-fold between the predictions and observations, respectively (no. 12 in Table 3). Most predicted values were generally within a half order of magnitude of observations for soil with pH < 8, while distinct over predictions were obtained for soil with pH > 8 (Figs 2 and 3). Simultaneously, better correlations between predictions and observations were obtained for the pH < 7 and pH 7–8 ranges, with R_adj_^2^ values being 0.92 and 0.91, respectively. The correlation was worse for pH > 8, with the value of R_adj_^2^ being 0.57. Similar results were observed for the leached soils. These results indicated that the precision levels in the prediction soluble Ni concentration were acceptable for soil pH < 8 and were unsatisfactory for soil pH > 8.

When the pH was > 8, NiCO_3_ (aq) was the most dominant soluble Ni species with fraction more than 40% and the proportion increased as pH increased [20]. The strong alkaline soils had a higher CaCO_3_ content and larger added dosages of Ni to the point that the NiCO_3_ (aq) may have oversaturated and precipitated on the soil. WHAM VI was unable to evaluate Ni precipitation in soils because the dissolution–precipitation reactions were not taken into account in the program. Similarly, Bonten et al. [28] concluded that the model tended to overestimate concentrations in solution for heavily contaminated soils because of potential precipitation. Therefore, it was not suitable to apply WHAM VI to evaluate soluble Ni concentrations in the calcareous and alkaline soils.

### 3.3. Visual MINTEQ Speciation

**Fig 4:**
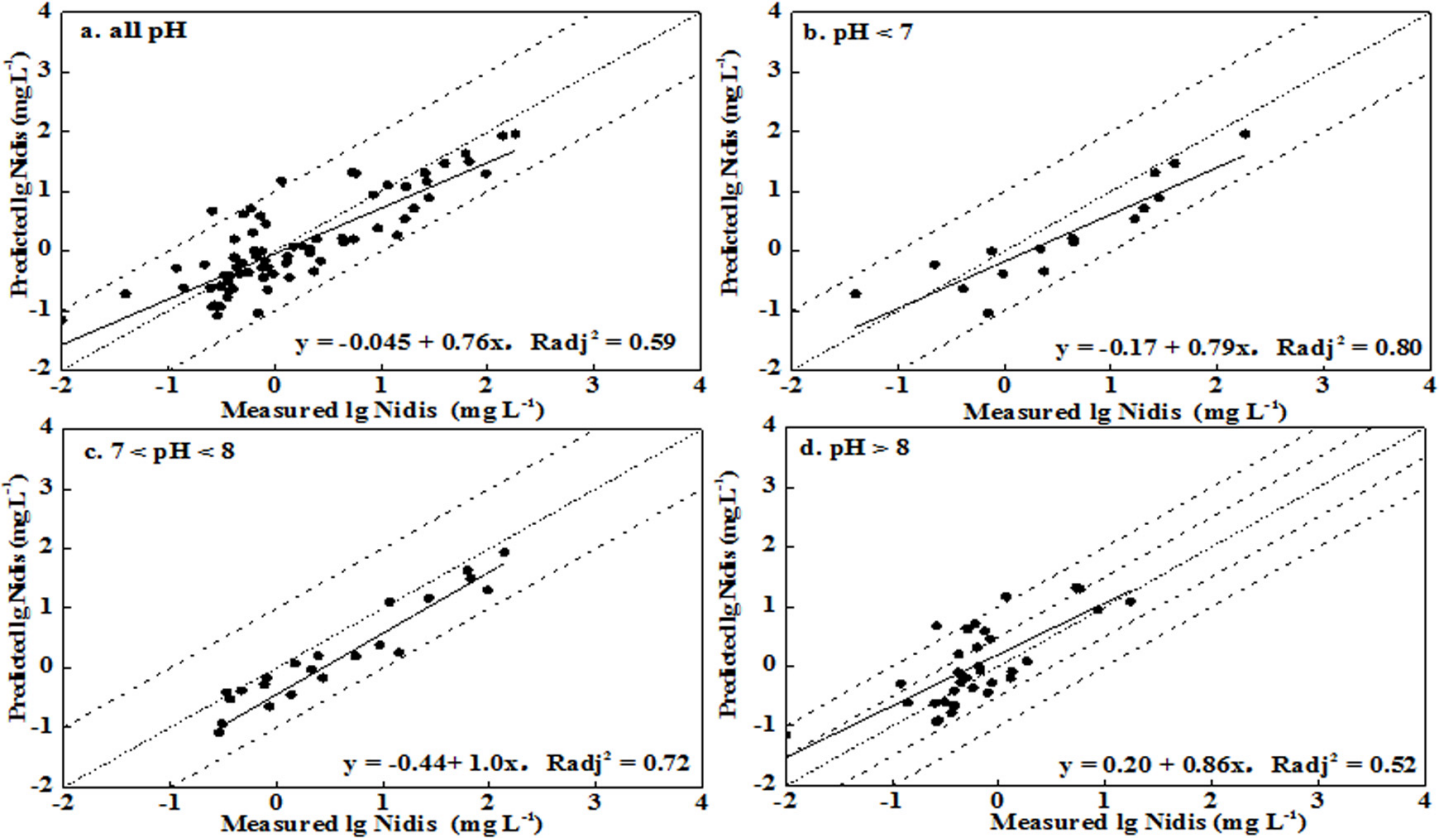
Measured soluble Ni concentration versus predicted Ni concentration using Visual MINTEQ for leached soils (Ni_dis_ represented the soluble Ni concentration in soil pore water).

**Fig 5:**
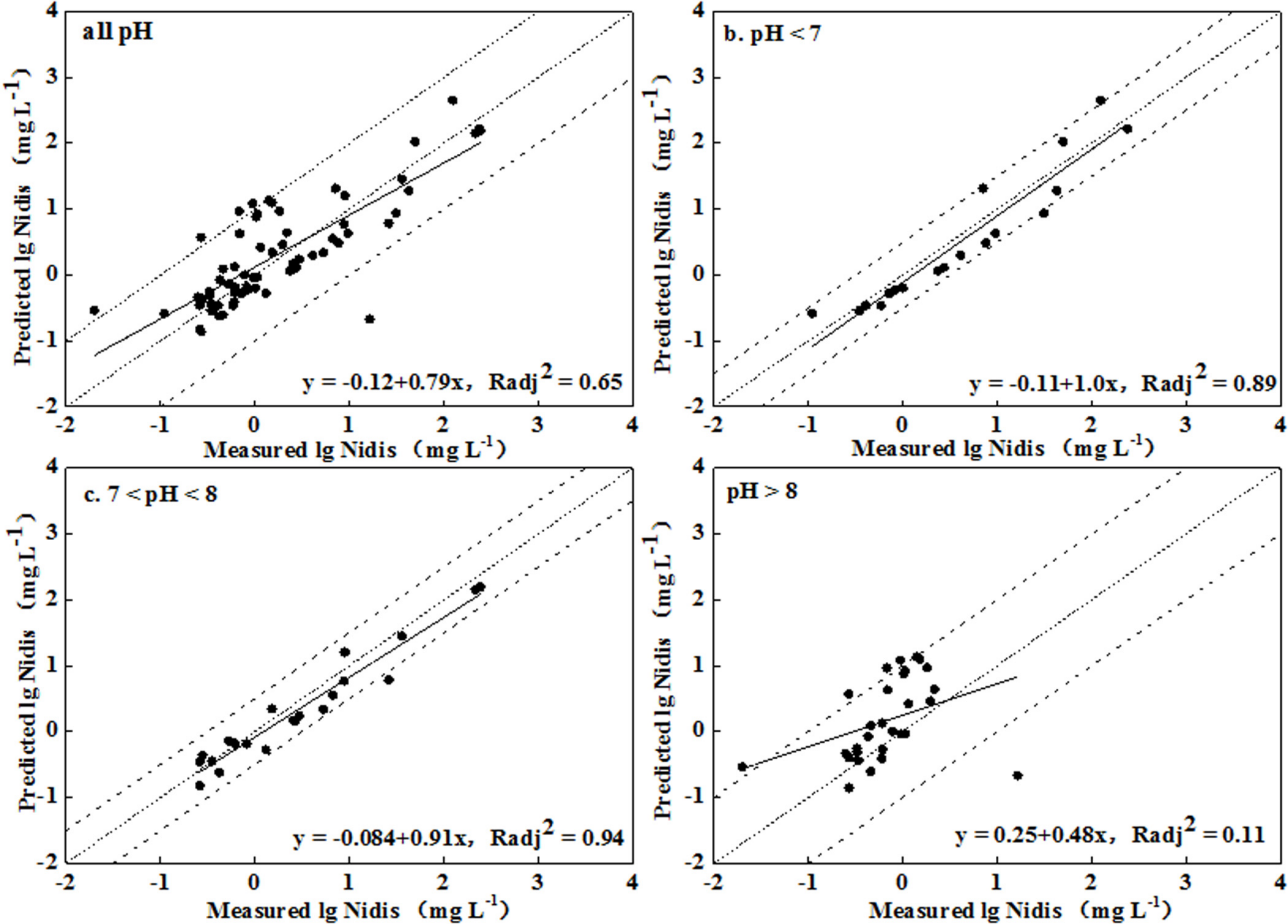
Measured soluble Ni concentration versus predicted Ni concentration using Visual MINTEQ in unleached soils (Ni_dis_ represented the soluble Ni concentration in soil pore water).

In order to advance the prediction of soil soluble Ni concentration, the other mechanistic model, Visual MINTEQ, was applied. The input parameters included: total soil Ni concentrations, SOM, pH, DOM, the activity of Al^3+^ and Fe^3+^, major dissolved anions, cations, and clay content. Since the soluble product of Al^3+^ and Fe^3+^ and the ratio of active DOM to DOC were the most important factors controlling the soluble Ni concentration, the model was optimized by adjusting these parameters. The results are shown in Table 4. Some differences were observed between the leached and unleached soils: the effects of dissolved Na^+^ and K^+^ could be ignored in model simulation for leached soils, while they could improve the prediction for unleached soils. Additionally, relatively better fits were obtained when the ratio of active DOM to DOC was 1.65 and 2.0 for leached and unleached soils, respectively. However, the SHM simulations in most cases fitted well with the observations from no. 1 and no. 10 for the leached and unleached soils, respectively, within an order of magnitude of observations (Figs 4 and 5). For soils with pH < 8, the model worked better for the unleached soils, with predictions within a half order of magnitude of observations. Meanwhile, better correlation between prediction and observation was obtained for soils with pH < 8, with R_adj_^2^ more than 0.89. For pH > 8, the RMSE values were 0.51 and 0.70 for the leached and unleached soil, respectively, corresponding to deviations of 3.2- and 5.0-fold between the predictions and observations. It was evident that the values were over predicted, which may have been caused by the original assumption of a high ratio of DOM to DOC. Although the deviation between the simulations and the observations was fairly large, the predictions were greatly improved by comparison with those from WHAM. These differences in model performance may be due to the greater number of adjustable parameters in the SHM, the determination of active DOM, a different set of soluble products for Al^3+^ and Fe^3+^, the possible precipitation calculation of NiCO_3_(s), and also other likely model-specific differences [13, 15].

**Table 4:** Effects of the input variables on the RMSE between the predicted soluble Ni concentrations by Visual MINTEQ and measured values.

No.	MINTEQ Inputs	RMSE (Leaching)				RMSE (Unleaching)			
		all	<7	7–8	>8	all	<7	7–8	>8
**1**	Ni_tot_+SOM+pH+Cation(K^+^, Na^+^, Ca^2+^, Mg^2+^)+anion (Cl^-^, SO_4_^2^)+DOC (DOM/DOC = 1.65)+Fe^3+^ (${p\;K}_{{Fe(OH)}_{3}}$ = 2.69) +Al^3+^ (${p\;K}_{{A\text{l}(OH)}_{3}}$ = 8.29)	0.49	0.49	0.58	0.51	0.50	0.37	0.29	0.67
**2**	Ni_tot_+SOM+pH+Cation(Ca^2+^,Mg^2+^)+anion (Cl^-^, SO_4_^2^)+DOC(DOM/DOC = 1.65)+Fe^3+^ (${p\;K}_{{Fe(OH)}_{3}}$ = 2.69) +Al^3+^ (${p\;K}_{{A\text{l}(OH)}_{3}}$ = 8.29)	0.56	0.50	0.68	0.51	0.54	0.44	0.36	0.69
**3**	Ni_tot_+SOM+pH+Cation (Ca^2+^, Mg^2+^)+anion (Cl^-^, SO_4_^2^)+DOC (DOM/DOC = 1.65)+Fe^3+^ (${p\;K}_{{Fe(OH)}_{3}}$ = 3.0) +Al^3+^ (${p\;K}_{{A\text{l}(OH)}_{3}}$ = 6.0)	0.92	0.88	1.20	0.71	-	-	-	-
**4**	Ni_tot_+SOM+pH+Cation (Ca^2+^, Mg^2+^)+anion (Cl^-^, SO_4_^2^)+DOC(DOM/DOC = 1.3)+Fe^3+^ (${p\;K}_{{Fe(OH)}_{3}}$ = 3.0) +Al^3+^ (${p\;K}_{{A\text{l}(OH)}_{3}}$ = 6.0)	0.58	0.55	0.74	0.47	-	-	-	-
**5**	Ni_tot_+SOM+pH+Cation(Ca^2+^, Mg^2+^)+anion (Cl^-^, SO_4_^2^)+DOC(DOM/DOC = 0.6)+Fe^3+^ (${p\;K}_{{Fe(OH)}_{3}}$ = 3.0) +Al^3+^ (${p\;K}_{{A\text{l}(OH)}_{3}}$ = 6.0)	0.73	0.77	0.96	0.51	-	-	-	-
**6**	Ni_tot_+SOM+pH+Cation (Ca^2+^, Mg^2+^)+anion (Cl^-^, SO_4_^2^)+DOC(DOM/DOC = 2)+Fe^3+^ (${p\;K}_{{Fe(OH)}_{3}}$ = 3.0) +Al^3+^ (${p\;K}_{{A\text{l}(OH)}_{3}}$ = 6.0)	0.56	0.46	0.63	0.55	-	-	-	-
**7**	Ni_tot_+SOM+pH+Cation (Ca^2+^, Mg^2+^)+anion (Cl^-^, SO_4_^2^)+DOC(DOM/DOC = 2)+Fe^3+^ (${p\;K}_{{Fe(OH)}_{3}}$ = 3.0) +Al^3+^ (${p\;K}_{{A\text{l}(OH)}_{3}}$ = 6.0) +Clay%	0.57	0.46	0.63	0.57	-	-	-	-
**8**	Ni_tot_+SOM+pH+Cation(K^+^, Na^+^, Ca^2+^, Mg^2+^)+anion(Cl^-^, SO_4_^2^)+DOC(DOM/DOC = 1.3)+Fe^3+^ (${p\;K}_{{Fe(OH)}_{3}}$ = 2.69) +Al^3+^ (${p\;K}_{{A\text{l}(OH)}_{3}}$ = 8.29)	-	-	-	-	0.51	0.43	0.36	0.64
**9**	Ni_tot_+SOM+pH+Cation(K^+^, Na^+^, Ca^2+^, Mg^2+^)+anion(Cl^-^, SO_4_^2^)+DOC(DOM/DOC = 0.6)+Fe^3+^ (${p\;K}_{{Fe(OH)}_{3}}$ = 2.69) +Al^3+^ (${p\;K}_{{A\text{l}(OH)}_{3}}$ = 8.29)	-	-	-	-	0.64	0.66	0.61	0.65
**10**	Ni_tot_+SOM+pH+Cation (K^+^, Na^+^, Ca^2+^, Mg^2+^)+anion(Cl^-^, SO_4_^2^)+DOC(DOM/DOC = 2)+Fe^3+^ (${p\;K}_{{Fe(OH)}_{3}}$ = 2.69) +Al^3+^ (${p\;K}_{{A\text{l}(OH)}_{3}}$ = 8.29)	-	-	-	-	0.50	0.33	0.25	0.70
**11**	Ni_tot_+SOM+pH+Cation(K^+^, Na^+^, Ca^2+^, Mg^2+^)+anion (Cl^-^, SO_4_^2^)+DOC(DOM/DOC = 2)+Fe^3+^ (${p\;K}_{{Fe(OH)}_{3}}$ = 2.69) +Al^3+^ (* ${p\;K}_{{A\text{l}(OH)}_{3}}$ = 8.29) +Clay%	-	-	-	-	0.52	0.33	0.25	0.73

Ni_tot_: total Ni concentration in soil; SOM: soil organic matter; DOC: dissolved organic carbon; RMSE: root mean square error.

A number of additional problems were also present in the simulation. For example, the interactions between the solid and soluble phases were complicated, and the ion strength of inputs exceeded the limits, which resulted in the number of iterations extending beyond the allowed maximum. These problems happened more often for soils with pH less than 8 and these simulated values were adopted for further analysis. Nevertheless, the simulations by Visual MINTEQ were reasonable, which made for significant progress in prediction of soil soluble Ni concentration for the high pH soils. Hence, the model can provide a valuable reference for practical applications if the ion loading is in the specified maximum.

## Supporting Information

S1 Fig Measured soluble Ni concentration versus predicted Ni concentration in leached soils from regression Equations (a. lgNi_dis_ = 0.76 + 1.24lgNi_tot_—0.46pH; b. lgNi_dis_ = 20.33 + 1.85lgNi_tot_—4.53lgAl_oxi—_2.82lgFe_oxi_; c. lgNi_dis_ = -1.53 + 1.84lgNitot—0.87lgAl_oxi_; d. lgNi_dis_ = 5.66 + 0.79lgNi_tot_—0.53pH—3.22lgAl_oxi_ + 1.94lgFe_oxi_) (Ni_tot_ and Ni_dis_ represented total Ni concentration in soil and the soluble Ni concentration in soil pore water, respectively; Al_oxi_ and Fe_oxi_ represented amorphous Al and Fe oxides, respectively). (DOC)

S2 Fig Measured soluble Ni concentration versus predicted Ni concentration in unleached soils from regression Equations (a. lgNi_dis_ = -0.24 + 1.51lgNi_tot_—0.39pH; b. lgNi_dis_ = 37.83 + 2.0lgNi_tot_—4.25pH + 5.01lgFe_oxi_—20.03lgClay; c. lgNi_dis_ = 5.70 + 2.10lgNi_tot_—1.39pH; d. lgNi_dis_ = -3.45 + 1.11lgNi_tot_ + 0.85lgFe_oxi_—1.33lgClay) (Ni_tot_ and Ni_dis_ represented total Ni concentration in soil and the soluble Ni concentration in soil pore water, respectively; Al_oxi_ and Fe_oxi_ represented amorphous Al and Fe oxides, respectively). (DOC)

S1 Table Correlation matrix (Pearson correlation cofficient) between lgNi_dis_ concentration in leached soil pore water and lgNi_tot_ together with soil properties (n = 97) (Ni_tot_ and Ni_dis_ represented total Ni concentration in soil and the soluble Ni concentration in soil pore water, respectively). (DOC)

S2 Table Correlation matrix (Pearson correlation cofficient) between lgNi_dis_ concentration in unleached soil pore water and lgNi_tot_ in soil together with soil properties (n = 102) (Ni_tot_ and Ni_dis_ represented total Ni concentration in soil and the soluble Ni concentration in soil pore water, respectively). (DOC)

S3 Table Multiple regressions between lgNi_dis_ concentration in soil pore water and lgNi_tot_ in soil together with soil properties (Ni_tot_ and Ni_dis_ represented total Ni concentration in soil and the soluble Ni concentration in soil pore water, respectively). (DOC)

## References

[1] Fairbrother A, Wenstel RW, Sappington K, Wood W. Framework for metals risk assessment. Ecotoxicol. Environ. Saf. 2007; 68 (2): 145–227. 17889701 10.1016/j.ecoenv.2007.03.015

[2] Tipping E, Rieuwerts J, Pan G, Ashmore MR, Lofts S, Hill MTR, et al The solid-solution partitioning of heavy metals (Cu, Zn, Cd, Pb) in upland soils of England and Wales. Environ. Pollut. 2003; 125 (2): 213–225. 12810315 10.1016/s0269-7491(03)00058-7

[3] Sauvé S, Hendershot W, Allen H. Solid-solution partitioning of metals in contaminated soils: dependence on pH, total metal burden, and organic matter. Environ. Sci. Technol. 2000; 34(7): 1125–1131.

[4] Sauvé S, Manna S, Turmel MC, Roy AG, Courchesne F. Solid−Solution partitioning of Cd, Cu, Ni, Pb, and Zn in the organic horizons of a forest soil. Environ. Sci. Technol. 2003; 37(22): 5191–5196. 14655707 10.1021/es030059g

[5] Weng LP, Wolthoorn A, Lexmond TM, Temminghoff EJM, van Riemsdijk WH. Understanding the effects of soil characteristics on phytotoxicity and bioavailability of nickel using speciation models. Environ. Sci. Technol. 2004; 38 (1): 156–162. 14740731 10.1021/es030053r

[6] Gandois L, Probst A, Dumat C. Modelling trace metal extractability and solubility in French forest soils by using soil properties. Eur. J. Soil Sci. 2010; 61(2): 271–286.

[7] Tye AM, Young S, Crout NMJ, Zhang H, Preston S, Zhao FJ, et al Speciation and solubility of Cu, Ni and Pb in contaminated soils. Eur. J. Soil Sci. 2004; 55 (3): 579–590.

[8] Dijkstra JJ, Meeussen JCL, Comans RNJ. Leaching of heavy metals from contaminated soils: An experimental and modeling study. Environ. Sci. Technol. 2004; 38(16): 4390–4395. 15382869 10.1021/es049885v

[9] Nolan AL, Ma YB, Lombi E, McLaughlin MJ. Speciation and isotopic exchange ability of Ni in soil solution. J. Environ. Qual. 2009; 38(2): 485–492. 10.2134/jeq2006.0275 19202018

[10] Ponizovsky AA, Thakali S, Allen HE, Di Toro DM, Ackerman AJ. Effect of soil properties on copper release in soil solutions at low moisture content. Environ. Toxicol. Chem. 2006; 25 (3): 671–682. 16566151 10.1897/04-621r.1

[11] Thakali S, Allen HE, Di Toro DM, Ponizovsky AA, Rooney CP, Zhao FJ, et al A terrestrial biotic ligand model. 1. development and application to Cu and Ni toxicities to barley root elongation in soils. Environ. Sci. Technol. 2006; 40(22): 7085–7093. 17154020 10.1021/es061171s

[12] Parat C, Cornu JY, Schneider A, Authier L, Sapin-Didier V, Denaix L, et al Comparison of two experimental speciation methods with a theoretical approach to monitor free and labile Cd fractions in soil solutions. Anal. Chim. Acta. 2009; 648 (2): 157–161. 10.1016/j.aca.2009.06.052 19646578

[13] Sjöstedt CS, Gustafsson JP, Köhler SJ. Chemical equilibrium modeling of organic acids, pH, aluminum, and iron in Swedish surface waters. Environ. Sci. Technol. 2010; 44(22): 8587–8593. 10.1021/es102415r 20958024

[14] Ytreberg E, Jenny Karlsson J, Hoppe S, Eklund B, Ndungu K. Effect of organic complexation on copper accumulation and toxicity to the Estuarine Red Macroalga Ceramium tenuicorne: a test of the free ion activity model. Environ. Sci. Technol. 2011; 45 (7): 3145–3153. 10.1021/es1039166 21391651

[15] Gustafsson JP, Van Schaik JWJ. Cation binding in a mor layer: batch experiments and modelling. Eur. J. Soil Sci. 2003; 54 (2): 295–310.

[16] Gustafsson JP, Pechová P. Modeling Metal Binding to Soils: The role of natural organic matter. Environ. Sci. Technol. 2003; 37 (12): 2767–2774. 12854717 10.1021/es026249t

[17] Li B, Zhang HT, Ma YB, McLaughlin MJ. Influences of soil properties and leaching on nickel toxicity to barley root elongation. Ecotox. Environ. Safe.2011; 74 (3): 459–466. 10.1016/j.ecoenv.2010.10.021 21030088

[18] Oorts K, Ghesquiere U, Smolders E. Leaching and aging decrease nickel toxicity to soil microbial processes in soils freshly spiked with nickel chloride. Environ. Toxicol. Chem. 2007; 26(6): 1130–1138. 17571677 10.1897/06-533r.1

[19] Zarcinas BA, McLaughlin MJ, Smart MK. The effect of acid digestion technique on the performance of nebulization systems used in inductively coupled plasma spectrometry. Commun. Soil Sci. Plant Anal. 1996; 27(5–8): 1331–1354.

[20] Li B, Zhang X, Wang XD, Ma YB. Refining a biotic ligand model for nickel toxicity to barley root elongation in solution culture. Ecotox. Environ. Safe. 2009; 72(6): 1760–1766. 10.1016/j.ecoenv.2009.05.003 19481262

[21] Axe L, Trivedi P. Intraparticle surface diffusion of metal contaminants and their attenuation in Microporous Amorphous Al, Fe, and Mn Oxides. J. Colloid Interf. Sci. 2002; 247(2): 259–265. 10.1006/jcis.2001.8125 16290464

[22] Tipping E, Rey-Castro C, Bryan SE, Hamilton-Taylor J. Al(III) and Fe(III) binding by humic substances in freshwaters, and implications for trace metal speciation. Geochim. Cosmochim. Acta 2002; 66 (18): 3211–3224.

[23] Contin M, Mondini C, Leita L, De Nobili M. Enhanced soil toxic metal fixation in iron (hydr)oxides by redox cycles. Geoderma. 2007; 140 (1–2): 164–175.

[24] Buekers J, Van Laer L, Amery F, Van Buggenhout S, Maes A, Smolders E. Role of soil constituents in fixation of soluble Zn, Cu, Ni and Cd added to soils. Eur. J. Soil Sci. 2007; 58(6): 1514–1524.

[25] Shi ZQ, Peltier E, Sparks DL. Kinetics of Ni sorption in soils: roles of soil organic matter and Ni precipitation. Environ. Sci. Technol. 2012; 46(4): 2212–2219. 10.1021/es202376c 22283487

[26] Weng LP, Temminghoff EJM, Van Riemsdijk WH. Contribution of individual sorbents to the control of heavy metal activity in sandy soil. Environ. Sci. Technol. 2001; 35(22): 4436–4443. 11757598 10.1021/es010085j

[27] Tipping E, Berggren D, Mulder J. Modelling the solid–solution distributions of protons, aluminium, base cations and humic substances in acid soils. Eur. J. Soil Sci. 1995; 46(1): 77–94.

[28] Bonten LTC, Groenenberg JE, Weng LP, van Riemsdijk WH. Use of speciation and complexation models to estimate heavy metal sorption in soils. Geoderma. 2008; 146(1–2): 303–310.

