# Supplementary material for: Predicting Soluble Nickel in Soils Using Soil Properties and Total Nickel
Source: PLoS One. 2015 Jul 28;10(7):e0133920. doi: 10.1371/journal.pone.0133920 (PMC4517763; doi:10.1371/journal.pone.0133920)
Supplement: S1 Fig — (DOC) [file pone.0133920.s001.doc]

**Figures for measured soluble Ni concentration versus Ni concentration in soils predicted by regression equations**

In our study, the soil pH values ranged from 4.93 to 8.9. In order to determine the influence of soil pH on the soil solid-solution phase distribution of Ni, the multiple regressions were divided into three ranges: pH < 7, 7 < pH <8 and pH >8.

**S1 Figure.** Measured soluble Ni concentration versus predicted Ni concentration in leached soils from regression Equations (a. lgNi_dis_ = 0.76 + 1.24lgNi_tot_ - 0.46pH; b. lgNi_dis_ = 20.33 + 1.85lgNi_tot_ - 4.53lgAl_oxi_ - 2.82lgFe_oxi_; c. lgNi_dis_ = -1.53 + 1.84lgNitot - 0.87lgAl_oxi_; d. lgNi_dis_ = 5.66 + 0.79lgNi_tot_ - 0.53pH - 3.22lgAl_oxi_ + 1.94lgFe_oxi_) (Ni_tot_ and Ni_dis_ represented total Ni concentration in soil and the soluble Ni concentration in soil pore water, respectively; Al_oxi_ and Fe_oxi_ represented amorphous Al and Fe oxides, respectively ).
