# Supplementary material for: Predicting Soluble Nickel in Soils Using Soil Properties and Total Nickel
Source: PLoS One. 2015 Jul 28;10(7):e0133920. doi: 10.1371/journal.pone.0133920 (PMC4517763; doi:10.1371/journal.pone.0133920)
Supplement: S2 Fig — (DOC) [file pone.0133920.s002.doc]

**S2 Figure.** Measured soluble Ni concentration versus predicted Ni concentration in unleached soils from regression Equations (a. lgNi_dis_ = -0.24 + 1.51lgNi_tot_ - 0.39pH; b. lgNi_dis_ = 37.83 + 2.0lgNi_tot_ - 4.25pH + 5.01lgFe_oxi_ - 20.03lgClay; c. lgNi_dis_ = 5.70 + 2.10lgNi_tot_ - 1.39pH; d. lgNi_dis_ = -3.45 + 1.11lgNi_tot_ + 0.85lgFe_oxi_ - 1.33lgClay) (Ni_tot_ and Ni_dis_ represented total Ni concentration in soil and the soluble Ni concentration in soil pore water, respectively; Al_oxi_ and Fe_oxi_ represented amorphous Al and Fe oxides, respectively ).
