# Supplementary material for: Predicting Soluble Nickel in Soils Using Soil Properties and Total Nickel
Source: PLoS One. 2015 Jul 28;10(7):e0133920. doi: 10.1371/journal.pone.0133920 (PMC4517763; doi:10.1371/journal.pone.0133920)
Supplement: S1 Table — (DOC) [file pone.0133920.s003.doc]

**Tables for correlation of soil properties and soluble Ni concentration in pore water**

The correlation of soil properties and soluble Ni concentration in pore water (Ni_dis_) was performed. The soluble Ni concentrations are more likely to be associated with soil Ni concentration (Ni_tot_), pH, clay content, sand content and Fe and Al oxides in leached soils, while the week correlation was observed for sand content and Al oxides with Ni_dis_ in unleached soils.

**S1 Table.** Correlation matrix (Pearson correlation cofficient) between lgNi_dis_ concentration in leached soil pore water and lgNi_tot_ together with soil properties (n=97) (Ni_tot_ and Ni_dis_ represented total Ni concentration in soil and the soluble Ni concentration in soil pore water, respectively).

|  | **pH** | **lgTC** | **lgOC** | **lgNi_tot_** | **lgNi_dis_** | **lgEC** | **lgCEC** | **lgClay** | **lgSilt** | **lgSand** | **lgAl_ox_** | **lgFe_ox_** | **lgMn_ox_** |
| --- | --- | --- | --- | --- | --- | --- | --- | --- | --- | --- | --- | --- | --- |
| **pH** | 1 | 0.06 | -0.32* | 0.18 | -0.48** | -0.07 | -0.19 | -0.82** | -0.39** | 0.83** | -0.69** | -0.47** | -0.24 |
| **lgTC** |  | 1 | 0.84** | 0.24 | -0.02 | 0.52** | 0.60** | 0.16 | 0.21 | 0.05 | 0.51** | 0.46** | 0.32* |
| **lgOC** |  |  | 1 | 0.15 | 0.19 | 0.48** | 0.72** | 0.40** | 0.20 | -0.19 | 0.67** | 0.61** | 0.28* |
| **lgNi_tot_** |  |  |  | 1 | 0.58** | 0.06 | 0.17 | -0.11 | 0.02 | 0.15 | 0.01 | 0.05 | -0.02 |
| **lgNi_dis_** |  |  |  |  | 1 | -0.06 | 0.11 | 0.33 | 0.10 | -0.32* | 0.31* | 0.30* | 0.02 |
| **lgEC** |  |  |  |  |  | 1 | 0.44** | 0.47** | 0.52** | -0.18 | 0.48** | 0.41** | 0.42** |
| **lgCEC** |  |  |  |  |  |  | 1 | 0.40** | 0.37** | -0.21 | 0.61** | 0.55** | 0.61** |
| **lgClay** |  |  |  |  |  |  |  | 1 | 0.65** | -0.90** | 0.86** | 0.57** | 0.51** |
| **lgSilt** |  |  |  |  |  |  |  |  | 1 | -0.66** | 0.58** | 0.52** | 0.64** |
| **lgSand** |  |  |  |  |  |  |  |  |  | 1 | -0.73** | -0.56** | -0.43** |
| **lgAl_ox_** |  |  |  |  |  |  |  |  |  |  | 1 | 0.60** | 0.68** |
| **lgFe_ox_** |  |  |  |  |  |  |  |  |  |  |  | 1 | 0.26 |
| **lgMn_ox_** |  |  |  |  |  |  |  |  |  |  |  |  | 1 |

EC: electric conductivity; eCEC: effective cation exchange capacity; TC: total carbon; OC: organic carbon; *:1%significant level,**: 1‰significant level.
