# Supplementary material for: Predicting Soluble Nickel in Soils Using Soil Properties and Total Nickel
Source: PLoS One. 2015 Jul 28;10(7):e0133920. doi: 10.1371/journal.pone.0133920 (PMC4517763; doi:10.1371/journal.pone.0133920)
Supplement: S2 Table — (DOC) [file pone.0133920.s004.doc]

**S2 Table.** Correlation matrix (Pearson correlation cofficient) between lgNi_dis_ concentration in unleached soil pore water and lgNi_tot_ in soil together with soil properties (n=102) (Ni_tot_ and Ni_dis_ represented total Ni concentration in soil and the soluble Ni concentration in soil pore water, respectively).

|  | **pH** | **lgTC** | **lgOC** | **lgNi_tot_** | **lgNi_dis_** | **lgEC** | **lgCEC** | **lgClay** | **lgSilt** | **lgSand** | **lgAl_ox_** | **lgFe_ox_** | **lgMn_ox_** |
| --- | --- | --- | --- | --- | --- | --- | --- | --- | --- | --- | --- | --- | --- |
| **pH** | 1 | 0.04 | -0.34** | 0.20 | -0.32** | -0.10 | -0.19 | -0.82** | -0.37** | 0.81** | -0.69** | -0.47** | -0.22 |
| **lgTC** |  | 1 | 0.84** | 0.19 | -0.02 | 0.51** | 0.59** | 0.15 | 0.24 | 0.04 | 0.52** | 0.46** | 0.30* |
| **lgOC** |  |  | 1 | 0.11 | 0.14 | 0.47** | 0.71** | 0.40** | 0.22 | -0.20 | 0.66** | 0.63** | 0.25 |
| **lgNi_tot_** |  |  |  | 1 | 0.72** | 0.05 | 0.09 | -0.12 | -0.09 | 0.18 | -0.02 | -0.01 | -0.08 |
| **lgNi_dis_** |  |  |  |  | 1 | 0.00 | 0.04 | 0.21 | 0.04 | -0.18 | 0.17 | 0.22 | -0.08 |
| **lgEC** |  |  |  |  |  | 1 | 0.43** | 0.49** | 0.57** | -0.22 | 0.50** | 0.42** | 0.40** |
| **lgCEC** |  |  |  |  |  |  | 1 | 0.37** | 0.38** | -0.19 | 0.59** | 0.53** | 0.60** |
| **lgClay** |  |  |  |  |  |  |  | 1 | 0.65** | -0.90** | 0.85** | 0.56** | 0.48** |
| **lgSilt** |  |  |  |  |  |  |  |  | 1 | -0.65** | 0.59** | 0.51** | 0.64** |
| **lgSand** |  |  |  |  |  |  |  |  |  | 1 | -0.73** | -0.55** | -0.43** |
| **lgAl_ox_** |  |  |  |  |  |  |  |  |  |  | 1 | 0.59** | 0.66** |
| **lgFe_ox_** |  |  |  |  |  |  |  |  |  |  |  | 1 | 0.22 |
| **lgMn_ox_** |  |  |  |  |  |  |  |  |  |  |  |  | 1 |

EC: electric conductivity; eCEC: effective cation exchange capacity; TC: total carbon; OC: organic carbon;italic numbers: *:1%significant level, **: 1‰ significant level
