# Supplementary material for: Predicting Soluble Nickel in Soils Using Soil Properties and Total Nickel
Source: PLoS One. 2015 Jul 28;10(7):e0133920. doi: 10.1371/journal.pone.0133920 (PMC4517763; doi:10.1371/journal.pone.0133920)
Supplement: S3 Table — (DOC) [file pone.0133920.s005.doc]

**S3 Table.** Multiple regressions between lgNi_dis_ concentration in soil pore water and lgNi_tot_ in soil together with soil properties (Ni_tot_ and Ni_dis_ represented total Ni concentration in soil and the soluble Ni concentration in soil pore water, respectively).

| **pH** | **No.** | **Constant** | **lgNi_tot_** | **pH** | **lgClay** | **lgFe** | **lgAl** | **lgSand** | **n** | **R^2^** | **R_adj_^2^** | **P** |
| --- | --- | --- | --- | --- | --- | --- | --- | --- | --- | --- | --- | --- |
| **Leaching-lgNi_dis_** | | | | | | | | | | | | |
| **All** | 1 | -2.24 | 1.05 |  |  |  |  |  | 97 | 0.34 | 0.33 | *** |
| **All** | 2 | 0.76 | 1.24 | -0.46 |  |  |  |  | 97 | 0.69 | 0.68 | *** |
| **All** | 3 | 4.36 | 1.30 | -0.59 |  |  | -0.95 |  | 97 | **0.71** | 0.71 | ** |
| **<7** | 4 | -2.04 | 1.23 |  |  |  |  |  | 30 | 0.42 | 0.40 | *** |
| **<7** | 5 | 3.47 | 1.61 |  |  | -1.87 |  |  | 30 | 0.73 | 0.71 | ** |
| **<7** | 6 | 20.33 | 1.85 |  |  | -2.82 | -4.53 |  | 30 | 0.90 | 0.88 | *** |
| **7~8** | 7 | -4.19 | 1.85 |  |  |  |  |  | 26 | 0.88 | 0.87 | *** |
| **7~8** | 8 | -1.53 | 1.84 |  |  |  | -0.87 |  | 26 | 0.91 | 0.90 | ** |
| **>8** | 9 | -1.86 | 0.68 |  |  |  |  |  | 41 | 0.40 | 0.39 | *** |
| **>8** | 10 | 0.41 | 0.70 |  | -1.85 |  |  |  | 41 | 0.58 | 0.56 | *** |
| **>8** | 11 | -2.34 | 0.67 |  | -2.36 | 1.21 |  |  | 41 | 0.74 | 0.72 | *** |
| **>8** | 12 | 0.16 | 0.77 |  |  | 1.75 | -2.68 |  | 41 | 0.76 | 0.74 | *** |
| **>8** | 13 | 5.66 | 0.79 | -0.53 |  | 1.94 | -3.22 |  | 41 | 0.80 | 0.78 | * |
| **>8** | 14 | 16.83 | 0.81 | -0.88 |  | 2.38 | -5.23 | -2.22 | 41 | 0.83 | 0.80 | * |
| **Unleaching-lgNi_dis_** | | | | | | | | | | | | |
| **All** | 15 | -2.77 | 1.33 |  |  |  |  |  | 102 | 0.52 | 0.52 | *** |
| **All** | 16 | -0.24 | 1.51 | -0.39 |  |  |  |  | 102 | 0.75 | 0.75 | *** |
| **All** | 17 | 2.88 | 1.53 | -0.57 | -1.29 |  |  |  | 102 | 0.77 | 0.77 | ** |
| **<7** | 18 | -2.55 | 1.47 |  |  |  |  |  | 32 | 0.56 | 0.55 | *** |
| **<7** | 19 | 1.48 | 1.64 |  |  | -1.29 |  |  | 32 | 0.70 | 0.68 | *** |
| **<7** | 20 | 10.24 | 1.69 |  | -3.74 | -2.07 |  |  | 32 | 0.76 | 0.73 | * |
| **<7** | 21 | 37.83 | 2.00 | -4.25 | -20.03 | 5.01 |  |  | 32 | 0.89 | 0.88 | *** |
| **7~8** | 22 | -4.47 | 2.02 |  |  |  |  |  | 28 | 0.86 | 0.85 | *** |
| **7~8** | 23 | 5.70 | 2.10 | -1.39 |  |  |  |  | 28 | 0.95 | 0.94 | *** |
| **7~8** | 24 | 4.42 | 2.08 | -1.74 |  | 1.23 |  |  | 28 | 0.96 | 0.96 | ** |
| **>8** | 25 | -2.71 | 1.12 |  |  |  |  |  | 42 | 0.73 | 0.72 | *** |
| **>8** | 26 | -1.48 | 1.13 |  | -0.98 |  |  |  | 42 | 0.76 | 0.75 | * |
| **>8** | 27 | -3.45 | 1.11 |  | -1.33 | 0.85 |  |  | 42 | 0.80 | 0.79 | ** |

Ni_dis_: soluble Ni concentration in soil pore water; Ni_tot_: total Ni concentration in soil; R^2^: coefficient of determination; R_adj_^2^: adjusted coefficient of determination; p: significant level of factors in regression equations; *: 5%significant level, **: 1%significant level, ***: 1‰significant level.
